# A Review of *FOXI3* Regulation of Development and Possible Roles in Cancer Progression and Metastasis

**DOI:** 10.3389/fcell.2018.00069

**Published:** 2018-07-03

**Authors:** Angana Mukherjee, Daniel P. Hollern, Oluwasina G. Williams, Tyeler S. Rayburn, William A. Byrd, Clayton Yates, Jacqueline D. Jones

**Affiliations:** ^1^Department of Biological Sciences, Troy University, Troy, AL, United States; ^2^Lineberger Comprehensive Cancer Center, University of North Carolina, Chapel Hill, NC, United States; ^3^Department of Biology and Center for Cancer Research, Tuskegee University, Tuskegee, AL, United States; ^4^Department of Nursing and Allied Health, Troy University, Troy, AL, United States

**Keywords:** *FOXI3*, embryogenesis, bone, development, cancer, metastasis

## Abstract

Development and cancer share a variety of functional traits such as EMT, cell migration, angiogenesis, and tissue remodeling. In addition, many cellular signaling pathways are noted to coordinate developmental processes and facilitate aspects of tumor progression. The Forkhead box superfamily of transcription factors consists of a highly conserved DNA binding domain, which binds to specific DNA sequences and play significant roles during adult tissue homoeostasis and embryogenesis including development, differentiation, metabolism, proliferation, apoptosis, migration, and invasion. Interestingly, various studies have implicated the role of key Fox family members such as *FOXP, FOXO*, and *FOXA* during cancer initiation and metastases. *FOXI3*, a member of the Forkhead family affects embryogenesis, development, and bone remodeling; however, no studies have reported a role in cancer. In this review, we summarize the role of *FOXI3* in embryogenesis and bone development and discuss its potential involvement in cancer progression with a focus on the bone metastasis. Moreover, we hypothesize possible mechanisms underlying the role of *FOXI3* in the development of solid tumor bone metastasis.

## Introduction

Embryogenesis, development, and solid tumor metastasis have many intersecting factors in regard to molecular events such as cell motility, changes in cellular differentiation, and changes in the interactions with cells of the local microenvironment (Kelleher et al., 2006; Heerboth et al., 2015). Indeed, these processes are tightly regulated and coordinated in part via gene regulation by transcription factors. Perhaps not surprisingly, a number of transcription factors have been shown to have overlapping roles in development, cancer progression, and cancer metastasis (Jackson et al., 1990; Schreiber et al., 2000; Hollern et al., 2014; Nowak et al., 2015; Zhang et al., 2016; To and Andrechek, 2018). Although some transcription factors have been well characterized for their roles in both development and in cancer; there remains many transcription factors with known significances in development that have not been examined for potential roles in carcinogenesis and metastatic progression. One such transcription factor with documented roles in development is *FOXI3*. Despite regulation of cancer-related processes such as epithelial to mesenchymal transition (Edlund et al., 2014; Shirokova et al., 2016); *FOXI3* remains unexplored in tumorigenesis and metastasis. In this review, we summarize the role of *FOXI3* in embryogenesis and bone development and examine its potential role in cancer progression using an informatics approach. Moreover, we discuss possible gene targets that might allow *FOXI3* to facilitate solid tumor bone metastasis.

## *FOXI3* in embryogenesis and development

The transcription factors of the Forkhead box family of transcription factors consist of a highly conserved, 110-amino acid Forkhead or “winged helix” DNA binding domain (DBD) which usually binds to DNA through RYAAAYA motif (where R = purine and Y = pyrimidine) (Nakagawa et al., 2013; Chen et al., 2016). The *FOX* genes of this family play important roles in various biological processes including embryogenesis, development and metabolism (Lehmann et al., 2003; Coffer and Burgering, 2004; Tuteja and Kaestner, 2007a,b).

### *FOXI3* in early bone development

Similar to other *FOX* genes, *FOXI3* plays a significant role in embryogenesis/development. In particular, a number of studies detail the *FOXI3* transcription factor as a critical regulator of bone development (Jänicke et al., 2007; Janicke et al., 2010; Drogemuller et al., 2008; Khatri and Groves, 2013; Khatri et al., 2014). The formation of skeletal bone tissue takes place during development where bones are formed by intramembranous and endochondral ossification. The latter process is dependent upon mesenchymal progenitor cells; marked by local concentration and differentiation initially into cartilage-forming chondrocytes which generate an avascular template on which new bone is formed (Regard et al., 2012). Upon completion of the skeletal system, bone becomes a regenerative tissue and is maintained by continuous remodeling (Regard et al., 2012). Implicating *FOXI3* in bone development, studies have shown that *FOXI3* induces early bone and cartilage formation. For instance, proper pharyngeal arch, and otic placode development was shown to be dependent upon *FOXI3* (Urness et al., 2010; Edlund et al., 2014; Khatri et al., 2014). Further, other work demonstrated *FOXI3* expression during early craniofacial development and showed that *FOXI3* maintains dental epithelial cells in an undifferentiated state (Jussila et al., 2015).

Indeed, additional studies point to wide ranging functions of *FOXI3* in development as a number of phenotypes have been observed with loss of *FOXI3* function. For example, mutation of *FOXI3* in canines result in abnormalities such as dysplastic hair follicles, complete absence of the pinna, absence of middle-ear structures, and malformed teeth (Drogemuller et al., 2008; Wiener et al., 2013; Tassano et al., 2015). In addition, mutations of *FOXI3* in humans result in Congenital Aural Atresia (CAA) (Tassano et al., 2015); a condition attributed to malformation of the outer ear. These findings demonstrate that *FOXI3* is a critical regulator of early cartilage and bone development (Drogemuller et al., 2008; Wiener et al., 2013). While it is clear that *FOXI3* is a key regulator of craniofacial development, there remains a gap in our knowledge of the mechanisms by which *FOXI3* operates in these processes, which cell-types utilize *FOXI3* to control development of cartilage and skeletal tissue and the genes the *FOXI3* regulates to govern these processes.

**Figure 1:**
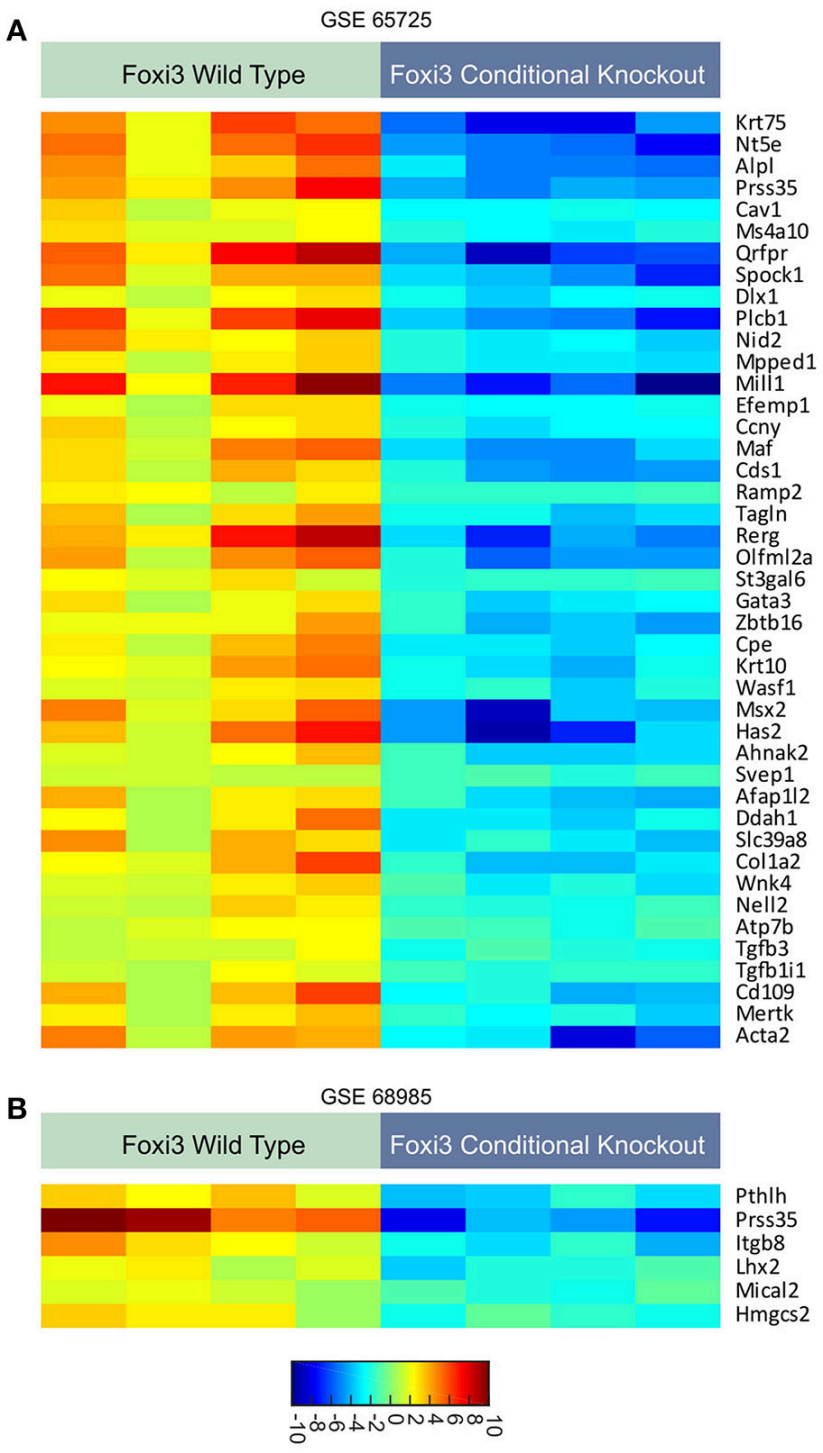
**(A,B)** Expression patterns of genes regulated with loss of *FOXI3*. Data from two published studies (Jussila et al., 2015; Shirokova et al., 2016) (accession numbers: GSE65725 and GSE68985) following download of series matrix files, data median centered, column standardized, and significant gene expression changes were identified using significant analysis of microarray (SAM) (Tusher et al., 2001). All depicted genes had a *q*-value < 0.0001. For both dataset, the FDR was minimized to 0.00%. The scale bar indicates the expression values corresponding to color.

Providing insight to these questions, recent work has uncovered some of the target genes by which *FOXI3* controls development. For instance, several studies examined the transcriptional impact of *FOXI3* loss using gene expression microarrays (Jussila et al., 2015; Shirokova et al., 2016). Jussila et al. performed expression profiling on *FOXI3* knockout mice to identify *Fgf15, Sfrp5 Dusp6, Etv5, Shh*, and *Ptch1* that were upregulated with *FOXI3* loss (Jussila et al., 2015). Their work concluded that loss of *FOXI3* led to abnormal upregulation of genes of Fgf, Shh and Bmp pathways, which in turn resulted in epithelial dysmorphogenesis during tooth development (Jussila et al., 2015). In a separate study, microarray analysis identified gene expression changes resulting from *FOXI3* loss in hair follicles; key amongst their findings is that *FOXI3* was essential for stem cell maintenance (Shirokova et al., 2016). Reanalyzing the gene expression data from these two studies (Jussila et al., 2015; Shirokova et al., 2016), using significant analysis of microarray (SAM), we highlight the genes significantly downregulated by *FOXI3* loss (Figure 1). As shown in Table 1, many of these genes have critical roles in development. Examining other molecules, work by Edlund et al. demonstrated FGF8 as a key effector of *FOXI3* signaling. In their work, Edlund et al. show that *FOXI3* mutants lose expression of *FGF8* and that loss of ectodermal *FOXI3*-Fgf8 signaling results in apoptosis of the neural crest cells (Edlund et al., 2014). Indeed, this work sheds light onto some of the details of *FOXI3* regulation of craniofacial development and points to *FOXI3* as a key regulator of neural crest cell survival. Together these studies point to *FOXI3* as a critical regulator of developmental pathways tied to stem cell function. Perhaps related to the criticality of *FOXI3* in maintenance of a de-differentiated state, recent work has implicated *FOXI3* in the epithelial-mesenchymal transition (Khatri and Groves, 2013; Edlund et al., 2014; Khatri et al., 2014).

**Table 1:** Role of *FOXI3* signature genes in early development.

***FOXI3* signature gene symbols**	**Negative fold change**	**Q-value**	**Accession number**	**Implications in development**	**References**
Cav1	3.077057237	0	GSE65725	Required for thymocyte development, cochlear inner hair cell development and T cell homeostasis	A.Jha., et al. *Science signaling*, 2015 (PMID: 26486172) B.A. Brandt., et al. *The journal of neuroscience*, 2003 (PMID: 14645476)
Spock1	33.97310003	0	GSE65725	Implicated in murine development	S. Roll., et al. *Matrix biology*, 2006 (PMID: 16806869) F. Charbonnier., et al. *Mechanisms of development*, 2000 (PMID: 10640720)
Maf	31.49087568	0	GSE65725	Essential for endochondral bone development	H. E. MacLean., et al. *Developmental biology*, 2003 (PMID: 14512017)
Ramp2	1.493731184	0	GSE65725	Plays a significant role in bone development, endocrine development, and angiogenesis	M. Kadmiel., et al. *Molecular endocrinology*, 2011 (PMID: 21566080) Y. Ichikawa-Shindo., et al. *The journal of clinical investigation*, 2008 (PMID: 18097473)
Gata3	3.117080473	0	GSE65725	Implicated in development and maintenance of type 2 innate lymphoid cells expressing IL-7Rα	T. Hoyler., et al. *Immunity*, 2012 (PMID: 23063333) R. Yagi., et al. *Immunity*, 2014 (PMID: 24631153)
Wasf1	2.190182385	0	GSE65725	Implicated in dendritic spine development and modulates oocyte transcription during embryogenesis	J. Y. Sung., et al. *Proc Natl Acad Sci U S A*, 2008 (PMID: 18287015) K. V., et al. *Science*, 2013 (PMID: 23990560)
Col1a2	15.09160309	0	GSE65725	Regulates formation and activity of collagen during development and constitutes about 90% of the bone matrix	M. Ponticos., et al. Matrix biology, 2004 (PMID: 15062855) M. L. Sohaskey., et al. The journal of cell biology, 2010 (PMID: 20440000)
Tgfb3	1.005055429	0	GSE65725	Plays a pivotal role in differentiation of osteoblasts and overall bone development	M. Wu., G. Chen., and Y. P. Li. *Bone research*, 2016 (PMID: 27563484) G. Chen., C. Deng., and Y. P. Li. *International journal of biological sciences*, 2012 (PMID: 22298955)
Pthlh	5.147615864	0	GSE68985	Implicated in murine embryogenesis, regulates endochondral bone development by inhibiting chondrocyte differentiation	L. Guo., et al. *PLoS one*, 2012 (PMID: 22808183) E. Minina., et al. *Developmental cell*, 2002 (PMID: 12361605) R. Flottmann., et al. *European journal of human genetics*, 2016 (PMID: 26733284)

### *FOXI3* in EMT

Epithelial-mesenchymal transition (EMT) is a key process in embryogenesis, wound healing and cancer. In EMT, a variety of gene expression changes are observed that are typically associated with a de-differentiated cellular state, and cells become motile (Ye et al., 2015; Hollern et al., 2018). Certainly, this transition is a process key to ectodermal, mesodermal, endodermal, and neural crest development (Yang and Weinberg, 2008; Voulgari and Pintzas, 2009). Importantly, neural crest cells are of mesenchymal differentiation and express *FOXI3* (Achilleos and Trainor, 2012; Khatri and Groves, 2013; Edlund et al., 2014; Niibe et al., 2017). During EMT, *FOXI3* with other EMT genes plays a significant role in the development of the epibranchial placodes, inner ear, and pharyngeal arch derivatives (Khatri and Groves, 2013; Edlund et al., 2014; Khatri et al., 2014). Furthermore, during neurulation, neural crest cells undergo EMT and express many genes critical to ion channel formation; an integral part of neural cell differentiation (Takahashi and Okamura, 1998; Scarpa et al., 2015). Interestingly, *FOXI3* is essential to ionocyte specification and differentiation in zebrafish (Hsiao et al., 2007; Jänicke et al., 2007). Like ion channels of human neural crest cells, ionocytes in zebrafish regulate ion transport, maintains homeostasis, and facilitates cell differentiation during EMT (Hsiao et al., 2007; Jänicke et al., 2007; Janicke et al., 2010; Thermes et al., 2010; Li and Xiong, 2011). During development, *FOXI3* also functions as a molecular switch, which when turned on, converts cells to ionocytes, and when turned off, to non-keratinocytes (Jänicke et al., 2007). Therefore, *FOXI3* has multimodal roles during development and the potent role of *FOXI3* in cell differentiation insinuate us about its possible involvement in abnormal cell growth and migration.

## *FOXI3* in cancer progression

Given that many developmental pathways, transcription factors, and processes, are commonly implicated in cancer progression, we propose that *FOXI3* may also play an important role in cancer. Indeed, with governance of developmental features such as stem cell maintenance and involvement in EMT, *FOXI3* may enable resistance to chemotherapy or tumor metastasis (Anders and Carey, 2008; Chaffer et al., 2016). However, to date no studies have been published that demonstrate *FOXI3*'s role in tumor progression. As such, we have utilized a variety of public data to examine *FOXI3* across cancer types and test for association with key clinical measures of tumor progression.

**Figure 2:**
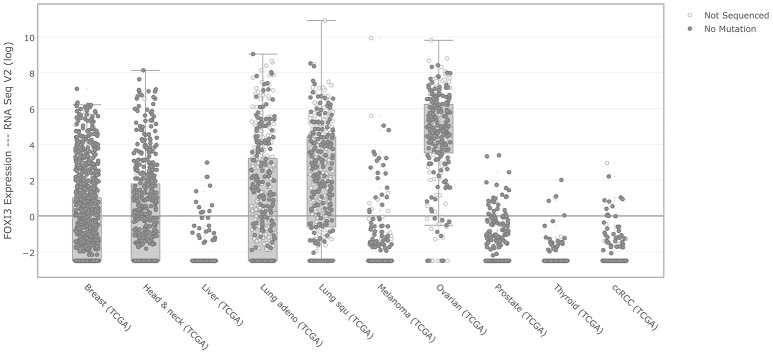
*FOXI3* expression across foremost cancer types. RSEM normalized gene expression levels of *FOXI3* as shown by cBioPortal (http://cbioportal.org) across TCGA cohorts.

One of the largest coordinated efforts to document the genomic features of cancer has been the work of The Cancer Genome Atlas. Using the cBioPortal to access multiple cancer types (Cerami et al., 2012; Gao et al., 2013), we first interrogated *FOXI3* expression across several major cancer types, (Figure 2). While relatively few liver and thyroid cancers show elevated *FOXI3* expression; other cancers display a wide range of *FOXI3* expression across patient tumors (breast, lung, prostate, ovarian, and head and neck cancer). With this, we questioned whether changes in *FOXI3* expression associate with features of tumor progression in several of these cancer types.

**Figure 3:**
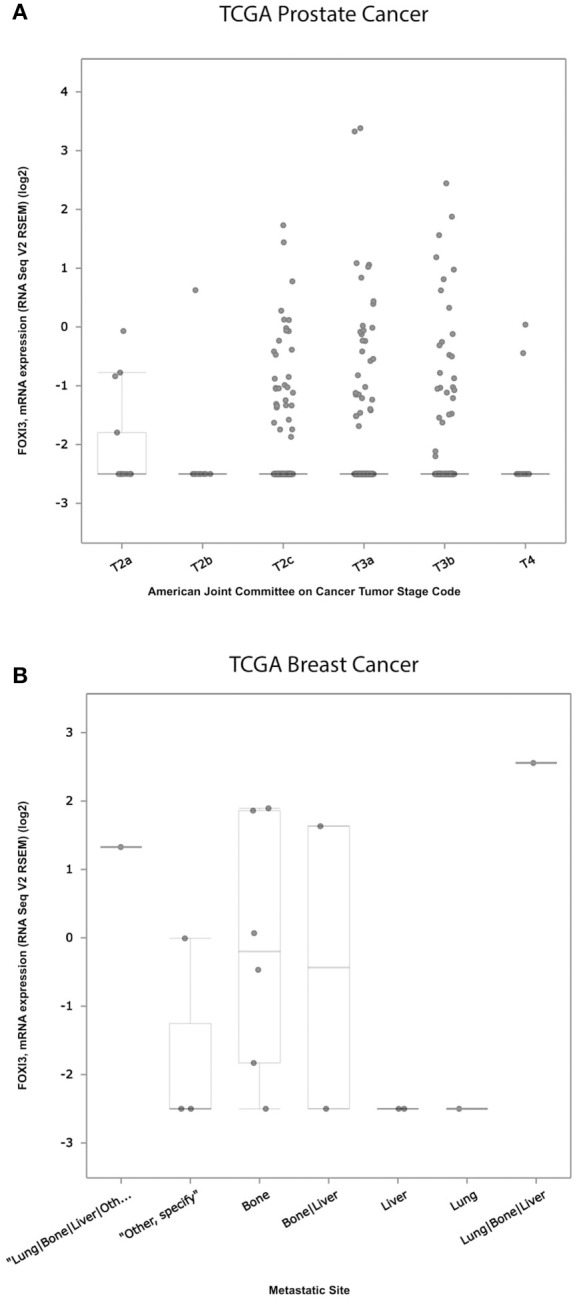
Expression of *FOXI3* in prostate and breast cancers. **(A)** RSEM normalized expression values of the TCGA prostate cancer cohort with tumor stage (Cancer Genome Atlas Research Network, 2015). **(B)** RSEM normalized expression values for TCGA breast tumors annotated according to site of distant metastasis (Anders and Carey, 2008). Both panels: Data was interrogated using cBioPortal (http://cbioportal.org).

Within the TCGA prostate cancer cohort (Cerami et al., 2012; Gao et al., 2013; Cancer Genome Atlas Research Network, 2015), we find that *FOXI3* expression increases with tumor stage (Figure 3A). While T4 tumors were infrequent in this cohort, many T3 stage tumors as compared to T2 stage have elevated *FOXI3* expression. The progression from T2 to T3 stage is classified in part by the presence of tumor cell invasion to the surrounding tissues of the prostate gland (T3) (Buyyounouski et al., 2017). This marks the first signs of metastatic potential of *FOXI3*. Indeed, given the bounty of research demonstrating EMT as critical to the invasive potential of prostate cancer, future research examining whether *FOXI3* expression functions in EMT, invasion, and metastasis could bring together these clinical associations to key processes of tumor progression controlled by *FOXI3*. Within the TCGA breast cancer cohort (Cancer Genome Atlas Research Network, 2012), some of the primary tumors were annotated for the site where distant metastasis was observed. Examining *FOXI3* expression in this data showed that breast cancers that metastasized to the bone often had high *FOXI3* expression (Figure 3B). While the number of cases are limited, additional experiments may illuminate the role of in facilitating bone metastasis. In support of this hypothesis, we noted that many of the genes shown to be downregulated with *FOXI3* loss (Figure 1), also have demonstrated roles in tumor progression and metastasis (Table 2), including bone metastasis.

**Table 2:** Role of *FOXI3* signature genes in cancer metastasis.

**FOXI3 signature gene symbols**	**Negative fold change**	***Q*-value**	**Accession number**	**Implications in cancer metastasis**	**References**
Cav1	3.077057237	0	GSE65725	Favors pancreatic cancer progression including invasion and migration to distant sites	M. Chatterjee., et al. *Scientific reports*, 2015 (PMID: 26065715).
Spock1	33.97310003	0	GSE65725	Promotes prostate cancer metastasis	Q. Chen., et al. *Drug design, development and therapy*, 2016 (PMID: 27486308)
Maf	31.49087568	0	GSE65725	Mediates breast cancer bone metastasis	M. Pavlovic., et al. *Journal of the cancer institute*, 2015 (PMID: 26376684)
Ramp2	1.493731184	0	GSE65725	Significantly promotes bone metastasis of breast cancer and is upregulated in prostate cancer	A. Cappariello., et al. Bone abstracts, 2013. M. Logan., et al. *The American journal of pathology*, 2013 (PMID: 23867798)
Gata3	3.117080473	0	GSE65725	Implicated in luminal breast cancer subtype	B. C. McCleskey., et al. *American journal of clinical pathology*, 2015 (PMID: 26486740)
Wasf1	2.190182385	0	GSE65725	Implicated in prostate cancer progression	H. S. Fernando., et al. *The journal of urology*, 2008 (PMID: 18710763)
Col1a2	15.09160309	0	GSE65725	Upregulated in gastric cancer and implicated in gastric cancer metastasis	C. Zhuo., et al. *Cell physiology and biochemistry*, 2016 (PMID: 27997896). N. Oue., et al. *Cancer research*, 2004 (PMID: 15059891)
Tgfb3	1.005055429	0	GSE65725	Associated with poor breast cancer prognosis. Implicated in prostate cancer metastasis. In bone metastasis, Tgfb triggers metastatic cancer cells to secrete factors in the bone microenvironment that in turn favors osteolytic bone destruction	A. Ghellal., et al. *Anticancer research*, 2000 (PMID: 11205281) X. Zhang., et al. *Experimental and therapeutic medicine*, 2016 (PMID: 27446297) J. T. Buijs., et al. *Bonekey reports*, 2012 (PMID: 23951484)
Pthlh	5.147615864	0	GSE68985	Promotes bone metastasis of breast cancer and nuclear PTHrP contributes to prostate cancer metastasis	K. Boras-Granic., and J. J. Wysolmerski*. Breast cancer research*, 2012 (PMID: 22546075) S. I. Park., and L. K. McCauley. *Endocrine-related cancer*, 2012 (PMID:22291434)

**Figure 4:**
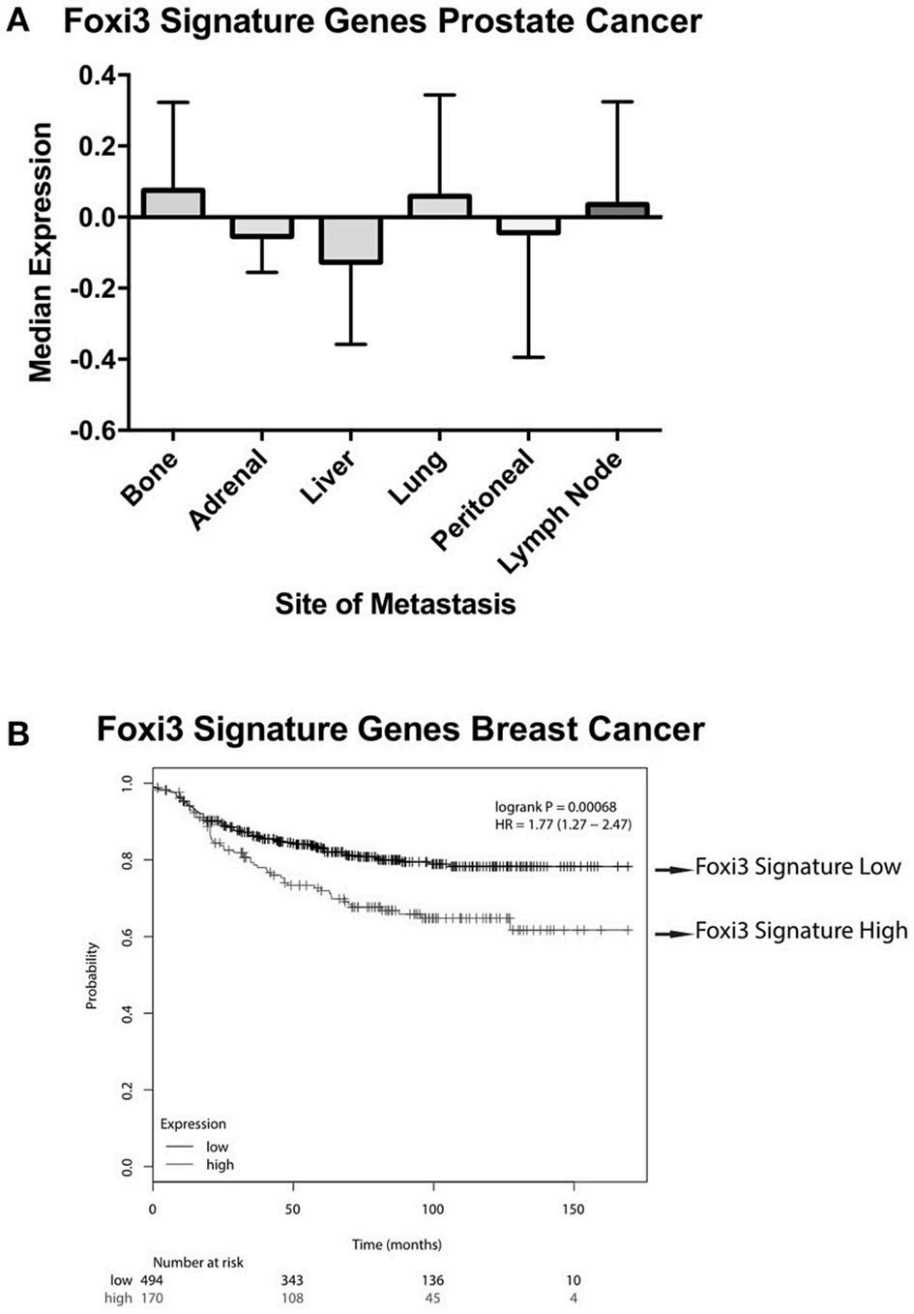
*FOXI3* signature genes in prostate and breast cancer metastasis. **(A)** Publicly available data (Haider et al., 2016) (accession number: GSE74685) downloaded from the series matrix file. Data was median centered and column standardized. Next, tumors were scored for median expression of FOXI3 signature genes depicted in Figure 1. The boxplots show median expression of these genes for tumors at distant sites of metastasis **(B)** Distant metastasis free survival is shown for breast cancer patient tumors scored for average expression of the *FOXI3* signature genes [*p*-value = 0.00068 and hazard ratio (HR) = 1.77 (1.27–2.47)]. Patient tumors were split into high and low expression groups using the autoselect threshold option on http://kmplot.com/analysis/ (Györffy et al., 2010).

These putative targets of *FOXI3* (Figure 1) also provide an additional tool for informatics inquiries. Initially, one of the limitations in our examination was the omission of probes for *FOXI3* on the microarrays of a variety of clinically rich gene expression datasets. However, the use of *FOXI3* putative target genes as a signature of *FOXI3* activity bypasses this limitation and allows for additional analyses. One dataset of particular interest was the published gene expression dataset across distant prostate cancer metastatic sites (Haider et al., 2016). As shown in Figure 4A, *FOXI3* signature levels were high in bone, lung, and lymph node metastases. *FOXI3* target genes also showed an association with metastasis in breast cancer. Probing expression data from primary breast tumors annotated for time to distant metastasis, we observed that tumors with the highest expression of the *FOXI3* signature genes present distant metastasis earlier than those with low expression (Figure 4B). Taken together with the observations in Figure 3A, *FOXI3* may mark and function in tumor cell invasion and metastasis. Given the clues from developmental studies, it might be that this function occurs by EMT and is an area that should be investigated. Additionally, data shown in Figures 3B, 4A, suggests that *FOXI3* may facilitate colonization of tumor cells to the bone. Indeed, a number of the putative targets of *FOXI3* have been implicated in bone metastasis and supports the need for future studies to examine *FOXI3* specifically in the bone metastasis setting.

## Implications for the future

**Figure 5:**
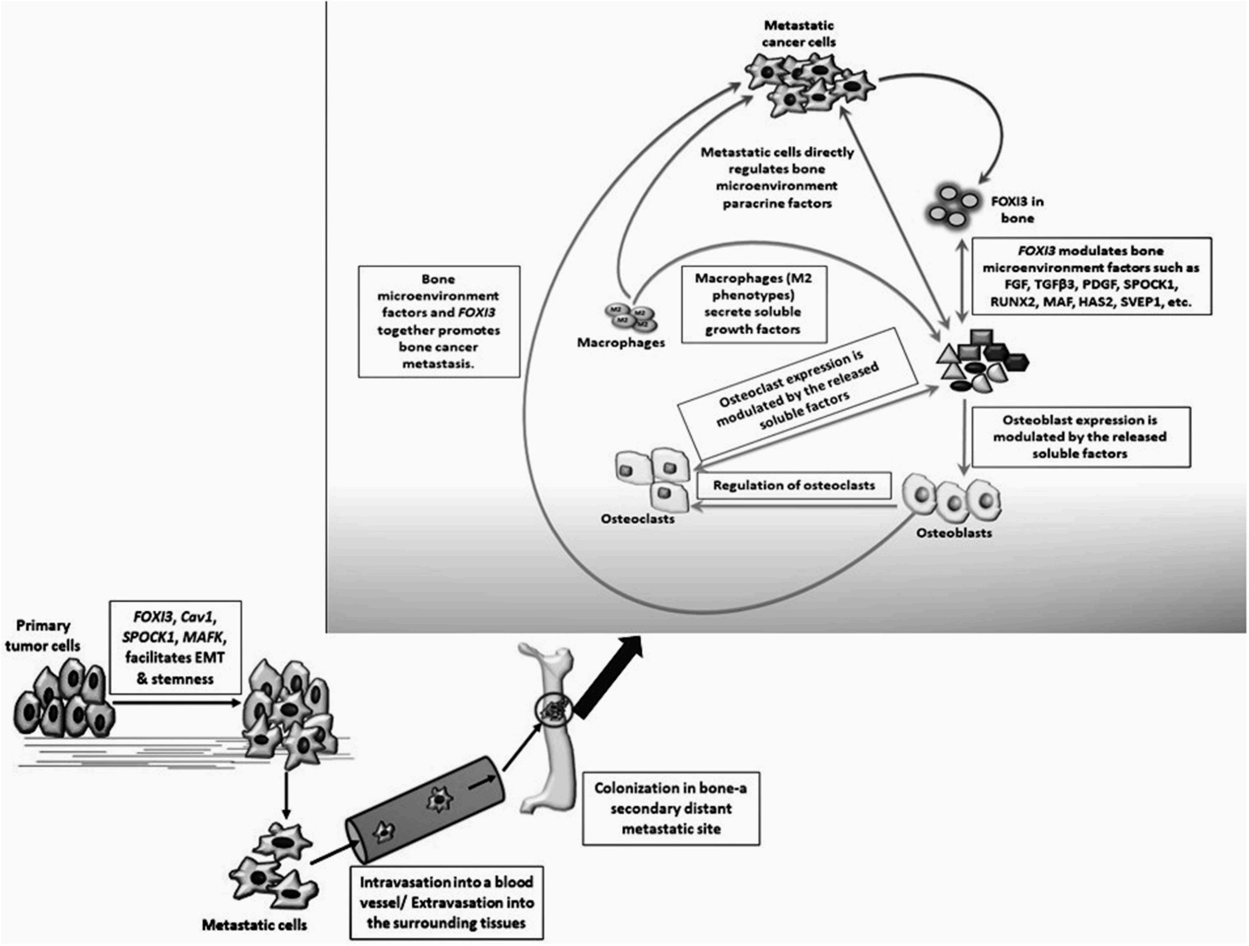
Hypothesized mechanisms for *FOXI3* role in cancer metastasis. FOXI3 may facilitate primary tumor cells to undergo EMT. Once the tumor cells invade the basement membrane, they enter the nearby blood or lymphatic vessel (intravasation) and tumors cells invade local microenvironment of distant sites via extravasation. Once these invasive migratory tumor cells colonize a fertile secondary tumor site such as bone, *FOXI3* may regulate the expression of bone matrix factors such as SPOCK1, TGFβ3, FGFs, and others to prepare the microenvironment in a way that aggravates cancer cell progression.

In this review, we note that *FOXI3* a regulator of early bone development may play a potential role in cancer progression (Table 3). We further note that several of the genes that are downregulated with the loss of *FOXI3*, such as SPOCK1, Dlx, MAF, PthrP, HAS2, SVEP1, and TGFβ3 (Jussila et al., 2015; Shirokova et al., 2016) have been shown to play important roles in distant metastasis to other sites including bone (Table 2). Based on our analysis of publicly available clinical data regarding *FOXI3* expression in different cancer types and stages, we speculate that *FOXI3* may have multiple roles throughout metastatic progression. At the primary tumor, *FOXI3* may allow for stem cell maintenance or EMT; the latter in turn may implicate *FOXI3* in tumor cell invasion into the local tissues and also into circulation. Further, in advanced cancer stage, during colonization of distant sites, *FOXI3* may facilitate reconfiguring of the tumor microenvironment by expression of paracrine factors. These mechanisms may come into play to facilitate bone metastasis as evidenced by several of the above listed potential target genes having previously demonstrated roles in bone metastasis (Table 2). Thus, we propose that when *FOXI3* in tumor cells modulate the secretion of other bone factors including SPOCK1, Dlx, MAF, PthrP, HAS2, SVEP1, TGFβ3, and FGF (Jussila et al., 2015; Meng et al., 2016; Shirokova et al., 2016) which further primes the microenvironment, creates a crosstalk to help tumors grow in the bone (Figure 5). Understanding the intersect of the regulatory behavior of *FOXI3* on factors prominent in hallmark processes of tumor progression to bone is essential to elucidating its role when these processes are hijacked and go awry. As a whole, this review highlights promising directions for research of *FOXI3* and its potential roles in cancer progression. Given no such studies exist to date, there is much to be discovered for this key developmental factor in the cancer setting and is a direction our lab plans to pursue.

**Table 3:** Summary describing the role of *FOXI3* in development and its hypothesized role in cancer progression.

**Processes**	**Role of FOXI3 in embryogenesis**	**Hypothesized role of FOXI3 in cancer**
Epithelial mesenchymal transition (EMT)	Early cartilage differentiation including development of epibranchial placodes, inner ear, and pharyngeal arch derivatives (Jussila et al., 2015)	May promote EMT, inducing metastasis
Bone development	**1**. Promotes murine craniofacial development (Jussila et al., 2015) **2**. Induces early bone and cartilage formation, i.e., pharyngeal and otic placode development in mice and chickens (Coffer and Burgering, 2004; Voulgari and Pintzas, 2009) **3**. Regulates ectodermal development in Chinese crested dogs and Mexican and Peruvian hairless dogs (Tuteja and Kaestner, 2007b; Shirokova et al., 2016).	Regulates bone matrix proteins, promoting osteomimicry of cancer cells, promoting bone metastasis
Bone microenvironment	**Fibroblast growth factor** **1**. Regulates FGF in vertebrate bone development (Khatri and Groves, 2013) **2**. Osteoclast/blast differentiation (Khatri and Groves, 2013) **Bone morphogenetic protein** Distal limb morphogenesis, mesoderm formation, and cell differentiation (Wiener et al., 2013; Tassano et al., 2015)	FGFs, SPOCK1, Dlx, RUNX2, MAF, PthrP, HAS2, SVEP1, TGFβ3, and other factors in bone matrix may modulate FOXI3 expression, promoting solid tumor bone metastasis
Ionocyte and ion channels	**1**. Regulates ionocyte differentiation in zebrafish embryos (Gao et al., 2013; Chen et al., 2016) **2**. Functions as a molecular switch converting cells to ionocytes (Chen et al., 2016)	Mutation of sub-cellular FOXI3A/B interferes with ion channel function, disrupting cell homeostasis and promoting tumor metastasis
Notch signaling	**Jagged-2** Regulates skin ionocyte formation and differentiation in zebrafish embryos by regulating Jagged-2 in the Notch Signaling Pathway (Gao et al., 2013; Chen et al., 2016)	FOXI3 overexpression may modulate Jagged-2, dysregulating the Notch pathway, promoting cancer invasion

## Author contributions

JJ and AM devised the concept with CY. AM, JJ, and OW developed the theory. DH, AM and OW developed and analyzed the tables. AM and DH, developed and interpreted the figures. AM, JJ, DH, OW, TR, WB, and CY drafted the manuscript.

### Conflict of interest statement

The authors declare that the research was conducted in the absence of any commercial or financial relationships that could be construed as a potential conflict of interest. The reviewer ADU and handling Editor declared their shared affiliation.
